# Interleukin-37 regulates innate immune signaling in human and mouse colonic organoids

**DOI:** 10.1038/s41598-021-87592-2

**Published:** 2021-04-15

**Authors:** Joannie M. Allaire, Anita Poon, Shauna M. Crowley, Xiao Han, Zohreh Sharafian, Navjit Moore, Martin Stahl, Brian Bressler, Pascal M. Lavoie, Kevan Jacobson, Xiaoxia Li, Bruce A. Vallance

**Affiliations:** 1 Department of Pediatrics, BC Children’s Hospital, University of British Columbia, Vancouver, BC Canada; 2 Division of Neonatology, Department of Pediatrics, BC Children’s Hospital, University of British Columbia, Vancouver, BC Canada; 3 STEMCELL Technologies, Vancouver, BC Canada; 4 Division of Gastroenterology, Department of Medicine, University of British Columbia, Vancouver, BC Canada; 5 Department of Immunology, Cleveland Clinic Foundation, Cleveland, OH USA

**Keywords:** Immunology, Chemokines, Cytokines, Cell biology, Extracellular signalling molecules

## Abstract

Intestinal epithelial cells (IEC) reside in close proximity to the gut microbiota and are hypo-responsive to bacterial products, likely to prevent maladaptive inflammatory responses. This is in part due to their strong expression of Single Ig IL-1 related receptor (SIGIRR), a negative regulator of interleukin (IL)-1 and toll-like receptor signaling. IL-37 is an anti-inflammatory cytokine that inhibits innate signaling in diverse cells by signaling through SIGIRR. Despite the strong expression of SIGIRR by IEC, few studies have examined whether IL-37 can suppress their innate immune signaling. We characterized innate immune responses of human and murine colonoids to bacteria (FliC, LPS) and host (IL-1β) products and the role of IL-37/SIGIRR in regulating these responses. We demonstrated that human colonoids responded only to FliC, but not to LPS or IL-1β. While colonoids derived from different donors displayed significant inter-individual variability in the magnitude of their innate responses to FliC stimulation, all colonoids released a variety of chemokines. Interestingly, IL-37 attenuated these responses through inhibition of p38 and NFκB signaling pathways. We determined that this suppression by IL-37 was SIGIRR dependent, in murine organoids. Along with species-specific differences in IEC innate responses, we show that IL-37 can promote IEC hypo-responsiveness by suppressing inflammatory signaling.

## Introduction

Intestinal epithelial cells (IEC) line the luminal surface of the mammalian gastrointestinal (GI) tract, and are uniquely positioned in that they are in constant and direct contact with both the gut microbiota as well as the underlying immune system. In this location, IEC play a central role in coordinating mucosal immunity, including promoting host defense by being responsive to luminal threats such as bacterial pathogens^1^. IEC accomplish this by expressing various pattern recognition receptors such as toll-like-receptors (TLR)^2–4^. These TLR enable IEC to sense those microbial pathogens that are able to escape the gut lumen and reach them, triggering IEC intrinsic pro-inflammatory and anti-microbial response, as well as signaling the presence of the invading microbes to underlying immune cells^4–6^. However, due to their frequent exposure to bacterial products released by commensal bacteria in the gut lumen under baseline conditions, IEC are less responsive to bacterial products than professional immune cells. This IEC hypo-responsiveness is likely aimed at preventing maladaptive inflammatory responses against the resident gut microbiota. This dichotomy suggests IEC undergo some form of dynamic innate immune regulation. Recent findings support this paradox by showing that human primary IEC are responsive to host (IL-1α, TNFα) and bacterial (flagellin) products, while being poorly responsive to other specific bacterial products such as LTA and LPS^7^. Another study showed that even though human IEC express the innate receptor for LPS (TLR4), they lack expression of the adaptor proteins necessary for proper activation of this innate receptor^2^.

Another way that IEC can regulate their own innate immune signaling is through the expression of negative regulators. We and others have shown that IEC strongly express Single Ig IL-1 related receptor (SIGIRR), a negative regulator of interleukin (IL)-1 and TLR signaling^8, 9^. Originally considered an orphan receptor, SIGIRR suppresses innate signaling by sequestering adaptor proteins downstream of MyD88, inhibiting intracellular signaling that would otherwise activate p38 MAP kinase or the transcription factor NFκB and their target genes^10, 11^. Previous studies using transformed IEC lines have shown that SIGIRR regulates NFκB -mediated chemokine (ie. IL-8) responses to IL-1β as well as TLR ligands (flagellin)^9^. Correspondingly, *Sigirr* deficient (−/−) mice have proven highly susceptible to intestinal inflammation and enteric infections, primarily due to the loss of *Sigirr* expression in their gut epithelium, leading to exaggerated inflammatory and antimicrobial responses^12, 13^. These studies have highlighted the importance of SIGIRR in controlling mucosal inflammation in the gut, yet its role as an orphan receptor has limited its potential to function as a therapeutic target.

This concept has changed since the discovery of IL-37, a newly discovered anti-inflammatory cytokine belonging to the IL-1 family^14^. In a variety of human diseases, elevated IL-37 levels have been linked with increased inflammation levels^15–17^. Specifically, IL-37 appears to play a key role in suppressing innate immunity, directly inhibiting inflammatory signaling in various cell types^18–20^. Interestingly, IL-37 is a human-specific cytokine and has not been discovered in mice. When human IL-37 is transgenically expressed in mice, it exhibits a wide range of anti-inflammatory features, acting through several mechanisms depending on the cellular and microenvironmental context^14, 21, 22^. For example, IL-37 can act intracellularly binding to SMAD3, translocating to the nucleus and directly suppressing the transcription of various cytokine and chemokine genes^14, 22^. In humans, IL-37 can also be produced and secreted extracellularly by immune cells^23^. Once in the extracellular environment, IL-37 binds to cells using a heterodimeric receptor comprised of both SIGIRR and the IL-18 receptor to inhibit innate immune signaling via specific inhibition of the NFκB and p38 MAPK pathways^21, 24^.

Despite IEC expressing higher levels of SIGIRR than other cells in the gut, thus far, no study has examined the ability of IL-37 and SIGIRR to regulate innate signaling within primary-derived IECs (ie. enteroids or colonoids). In this study, we evaluated the responsiveness of primary colonic IEC (colonoids), derived from healthy donors, to the pro-inflammatory cytokine IL-1β and to the microbial products flagellin (FliC) and LPS. Only the bacterial product FliC induced a strong inflammatory response in human colonoids, whereas these cells showed a very limited response to IL-1β as compared to transformed cell lines (Caco-2) or mouse colonoids. Although we observed significant inter-individual variability upon FliC stimulation, all colonoids responded by releasing several chemokines (IL-8, CCL2 and CCL20). In contrast, mouse colonoids were found to be responsive to both FliC and IL-1β but only secreted a limited number of chemokines (Cxcl1 and Ccl20). For the first time, we show that the anti-inflammatory cytokine IL-37 attenuates the innate immune responses of human and mouse colonoids and this attenuation, at least in murine cells was Sigirr dependent. Taken together, our results show exogenous IL-37 can suppress innate immune responses of primary IEC and that important inter-species differences in IEC responses to specific inflammatory stimuli occur.

## Results

### Pediatric and adult colonoids transcribe similar levels of innate immune genes

IL-37 expression and function have been intensively studied in immune cells such macrophages, neutrophils, and T cells, but little attention has been paid to epithelial cells. To better understand the innate immune response of primary intestinal epithelial cells (IEC) and whether it is impacted by IL-37, human-derived organoids were grown from the sigmoidal biopsies of pediatric or adult healthy donors according to well described methods^25, 26^. These colonoids were exposed to the cytokine IL-1β or bacterial products (LPS or flagellin (FliC)) for 4 h and then assessed for their immune response by qPCR, ELISA and Western Blot analysis. After being cultured for 10 to 12 days, both adult and pediatric colonoids showed similar macroscopic and histological features, including budding and IEC thickening (see Fig. S1 online). Since IL-37 typically acts under inflammatory conditions, expression of various innate receptors was evaluated in human colonoids to determine their capacity to respond to pro-inflammatory stimuli. Recently published studies have demonstrated site specific innate receptor expression as well as human specific innate responses by IEC to host or microbial products^2, 3, 27^. By real-time qPCR analysis, both pediatric and adult colonoids were confirmed to transcribe mRNA for several innate immune receptors including *TLR4*, *TLR5 IL-1R* and *IL-18R1* (see Fig. S1 online). Gene transcription of *SIGIRR* and the novel anti-inflammatory cytokine, *IL-37* were also confirmed by qPCR analysis, showing similar levels between pediatric and adult samples (see Fig. S1 online). Human colonoids showed high expression of *SIGIRR mRNA* but very low levels of IL-37. IL37 protein secretion was not detected in IEC under all stimulation conditions as opposed to LPS stimulated peripheral blood mononuclear cells (PBMC) that secreted IL-37 (see Fig. S1 online)^23^.

### Human colonoids are highly responsive to *Salmonella* flagellin

**Figure 1:**
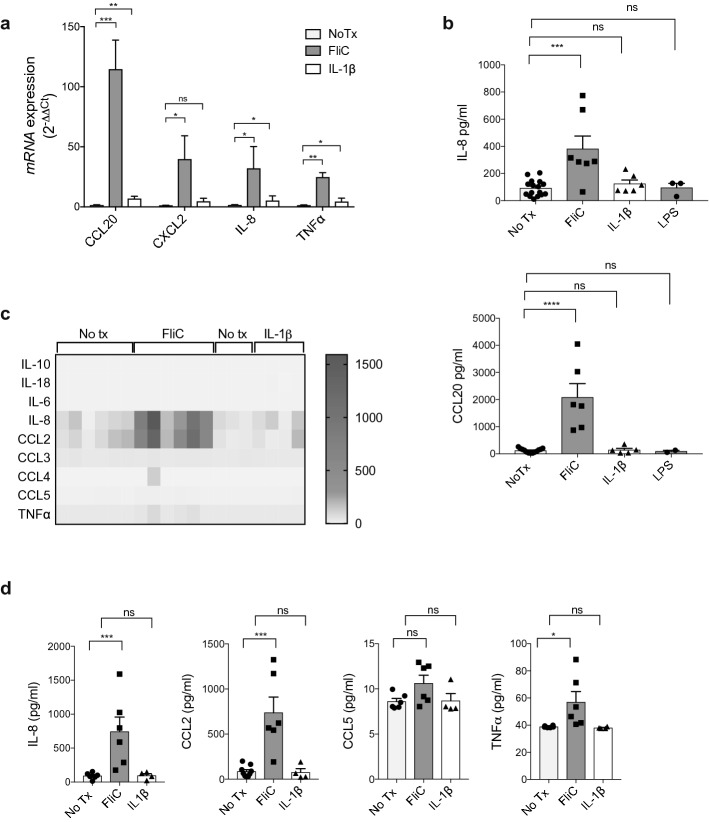
Human IEC responses to host (IL-1β) and bacterial stimuli (FliC, LPS). (**a**) qPCR analysis of inflammatory gene transcription after FliC or IL-1β stimulation for 4 h, expressed as fold change over untreated colonoids. (**b**) IL-8 (top) and CCL20 (bottom) protein levels secreted basolaterally by human colonoids after 4 h of stimulation with FliC or IL-1β. (**c**) Heat map representation of chemokine concentrations in the supernantants of colonoids after 4 h of stimulation with FliC or IL-1β. (**d**) Concentration of chemokines detected by Multiplex Luminex Assay in the supernatants of colonoids after 4 h of stimulation with FliC or IL-1β. Mean and SEM are indicated from n = 6–8 donors (4 adult and 2–4 pediatric). All data shown are representative of at least 2–3 independent experiments. Statistical significance calculated using unpaired Student’s *t*-test *, *P* = 0.01 to 0.05; **, *P* = 0.001 to 0.01; ***, *P* = 0.0001 to 0.001. ns = not significant.

To analyze the inflammatory response and functionality of naïve, primary IEC, mature 3D colonoids in Matrigel were treated with *Salmonella* flagellin (FliC 100 ng/ml) for 4 h. Colonoids were also treated with IL-1β (10 ng/ml) or the bacterial product LPS (1 µg/ml). FliC stimulation of colonoids elicited strong upregulated transcription of various cytokine and chemokine genes at the mRNA level, with *CCL20* induced 120-fold, and *IL-8* and *TNFa* induced 40-fold compared to untreated conditions (Fig. 1a). In contrast, IL-1β stimulation induced only a 4 to sixfold increase in mRNA levels of these cytokines (Fig. 1a). To determine if these changes led to protein secretion, we collected supernatant of stimulated 3D colonoids in Matrigel, to look at basolateral secretion, and performed ELISA. Consistent with mRNA, FliC, among the 3 stimuli used, was the strongest inducer of IEC innate responses with a strong CCL20 and IL-8 secretion, whereas IL-1β induced little or no secretion of these chemokines (Fig. 1b). The low or lack of innate response of human colonoids to IL-1β stimulation is not caused by the ineffectiveness of IL-1β, as IL-1β, even at a concentration of 5 times less, was able to induce higher levels of IL-8 and CCL20 secretion by Caco2 cells (see Fig. S1 online). LPS also failed to induce the secretion of IL-8 and CCL20 by human colonoids, similar to a recent finding^3^ (Fig. 1b).

One striking observation from the FliC stimulation experiments was the significant variability among the 6 colonoid lines (each derived from a different patient) in the magnitude of their innate immune response (Fig. 1b). CCL20 secretion by the different colonoid lines ranged from 200 to 1200 pg/ml after 4 h of FliC stimulation despite ensuring equal colonoid density, size and protein quantity. To explore the expression/secretion of other cytokines and chemokines the colonoid innate immune response to FliC and how this differed among individuals, we used a Milliplex Luminex assay detecting 9 cytokines and chemokines (IL-6, IL-10, IL-18, IL-8, CCL2, CCL3, CCL4, CCL5, TNFα) (Fig. 1c). Notably, FliC-stimulated colonoids produced CCL2 (MCP-1) at high levels similar to the levels detected for IL-8, followed by CCL5 and TNFα at more modest levels (Fig. 1d). ELISA also confirmed the high levels of IL-8 secreted (Fig. 1b). Furthermore, it is important to mention that under these specific conditions, IL-10, IL-6, IL-18, CCL3 and CCL4 secretion were not detected (Fig. 1c).

### IL-37 suppresses innate immune responses of human colonoids to different stimuli

**Figure 2:**
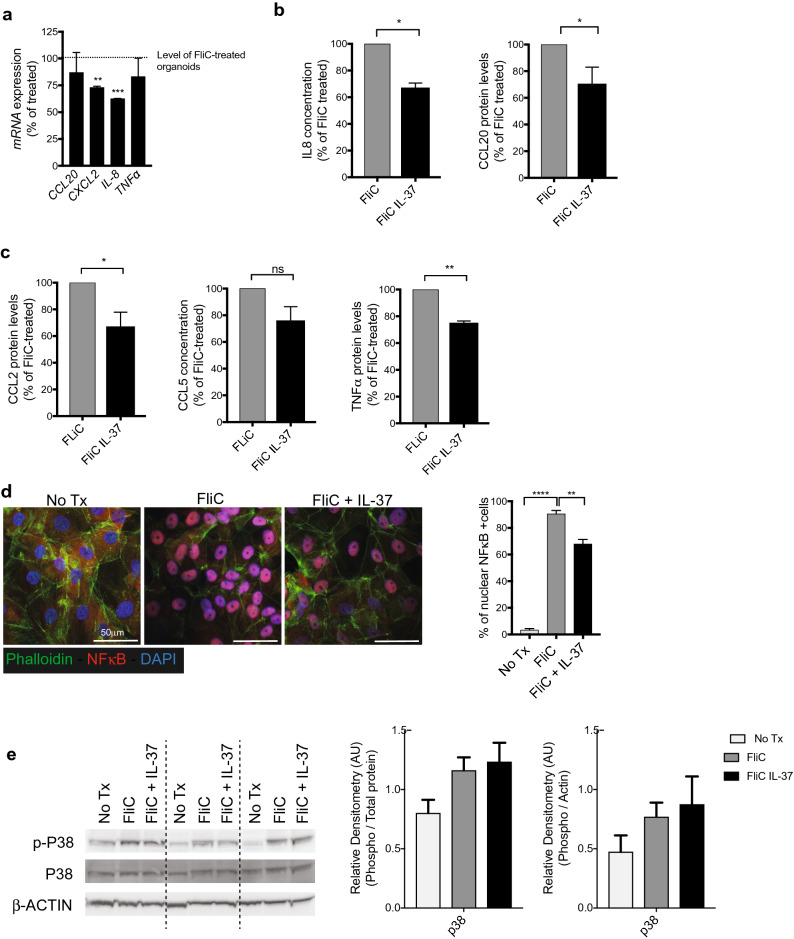
IL-37 effect on human IEC FliC responses. (**a**) qPCR analysis of inflammatory genes after FliC and IL-37 exposure for 4 h expressed as percentage of change over FliC treated colonoids. (**b**) IL-8 and CCL20 protein levels (detected by ELISA) secreted basolaterally by human colonoids after 4 h of stimulation with FliC and IL-37. (**c**) CCL2, CCL5 and TNFa protein levels secreted basolaterally by colonoids after 4 h of stimulation with FliC and IL-37 (detected by Milliplex Luminex assay). (**d**) Immunostaining against NFkB (red), actin (Phalloidin—green) and DAPI (blue) of 2D monolayer after 30 min of stimulation with FliC with or without IL-37 (left). Counts of NFkB positive nuclei from immunostaining (right). (**e**) Western blot analysis of phospho and total p38, after 30 min of stimulation with FliC with or without IL-37 (left). Equal loading confirmed with β-Actin as well as total protein stain of membrane (see Fig. S4 online). Densities relative to total protein or β-Actin are shown (right). Mean and SEM are indicated from n = 4 donors (2 adult and 2 pediatric). All data shown are representative of at least 3 independent experiments. Statistical significance calculated using unpaired Student’s *t*-test *, *P* = 0.01 to 0.05; **, *P* = 0.001 to 0.01; ***, *P* = 0.0001 to 0.001; ****, *P* = 0.00001 to 0.0001; ns = not significant.

We next determined if IL-37 would inhibit IEC immune responses. A dose response curve for IL-37 stimulation revealed that, much like peripheral blood mononuclear cells (PBMC)^21^, picogram levels of IL-37 (1000 pg/ml to 100 pg/ml) were sufficient to suppress FliC-induced inflammatory responses in colonoids (see Fig. S2 online).Therefore, 100 pg/ml of IL-37 was selected to evaluate its effect on innate immune responses of FliC stimulated colonoids. The mRNA levels of various cytokines and chemokines such as *CCL20*, *IL-8*, *CXCL2* and *TNF*α were significantly reduced in the presence of IL-37 (Fig. 2a). These mRNA changes were found to largely correlate with protein levels as demonstrated through ELISA (Fig. 2b) or Milliplex Luminex analysis (Fig. 2c). However, the majority of colonoids lines (4 of 6) showed a significant reduction of CCL20 and IL-8 secretion, while the colonoids from 2 individuals were unresponsive to IL-37 treatment (see Fig. S2 online). To further investigate the effects of IL-37, we pursued our analysis only on the IEC/colonoids derived from responsive donors. Among these responsive colonoids, a significant reduction was observed at the protein levels for IL-8 secretion (34% reduction) and CCL20 at 25% (Fig. 2b) as well as significant decrease in CCL2 and TNFα levels in response to IL-37 treatment (Fig. 2c).

### IL-37 dramatically suppresses FliC-induced activation of NFκB in human colonoids

In immune cells, the downstream immunosuppressive effects of IL-37 via SIGIRR are hypothesized to occur through intracellular inhibition of the NFκB or p38 MAPK signaling cascades, leading to reduced inflammatory signals including attenuated cytokine and chemokine secretion^21, 24^. To assess whether the effect of IL-37 observed in the IEC/colonoids was due to its suppression on p38 or NFκB activation, we took advantage of a 2D monolayer system derived from human colonoids to assess NFκB activation through immunostaining. This involved the dissociation of 3D colonoids and plating single cells onto an ECM-coated coverslip to form a monolayer^27–29^. After 5 days of culture, when confluency was well established, monolayers were treated with FliC (100 ng/ml) for 30 min to provide sufficient time for NFκB translocation to the nucleus, indicative of its activation (Fig. 2d). As expected, exposure to FliC led to the majority of NFκB migrating to the nuclei of the IEC. When the monolayers were also exposed to IL-37, significant reductions in both the number of cells with nuclear NFκB signal, and the intensity of the nuclear staining were observed (Fig. 2d). Surprisingly, western blot analysis showed that the activation and phosphorylation of p38 was only modestly increased after FliC stimulation and IL-37 did not reduce its activation level (Fig. 2e).

### IL-37 moderately suppresses FliC-induced p38 activation in human colonoids

**Figure 3:**
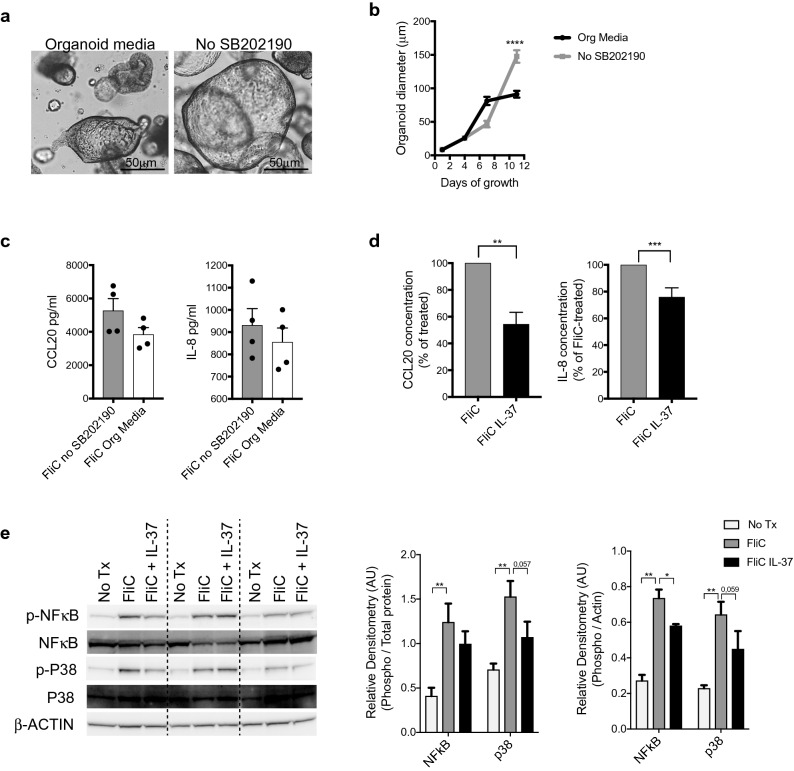
SB202190 effect on IL37 immunosuppressive responses. (**a**) Brightfield images of a representative human colonoids grown in standard organoid Media (left) or in No SB202190 media (right). **b**) Growth curve of colonoids grown in standard organoid media or in No SB202190 media for 12 days. (**c**) IL-8 and CCL20 protein levels secreted basolaterally after 4 h of stimulation with FliC of human colonoids grown in standard organoids media or in No SB202190 media. (**d**) IL-8 and CCL20 protein levels secreted basolaterally by human colonoids grown in No SB202190 media after 4 h of stimulation simultaneously with FliC and IL-37. (**e**) Western blot analysis of phospho and total NFkB, phospho and total p38 in human colonoids grown in No SB202 media after 30 min of stimulation with FliC with or without IL-37 (left). Equal loading confirmed with β-Actin as well as total protein stain of membrane (see Fig. S5 online). Densities relative to total protein or β-Actin are shown (right). Mean and SEM are indicated from n = 4 donors (2 adult and 2 pediatric). All data shown are representative of at least 3 independent experiments. Statistical significance calculated using unpaired Student’s *t*-test *, *P* = 0.01 to 0.05; **, *P* = 0.001 to 0.01; ***, *P* = 0.0001 to 0.001; ****, *P* = 0.00001 to 0.0001. Comparison lacking annotations are not significant.

SB202190 is an important media component necessary for the growth and proliferation of organoids, however it is also a potent inhibitor of p38 MAPK^26^. Even though the colonoids were treated in a serum-growth factor free medium (base medium; Advanced DMEM/F12 supplemented with GlutaMAX and HEPES), they were grown in the presence of SB202190 until their stimulation, which could impact their ability to respond to IL-37 and FliC. To address this, we grew colonoids in an organoid media lacking SB202190. There were alterations in the appearance and growth rate of the colonoids in the absence of SB202190 (Fig. 3a,b). The colonoids appeared more cystic and less mature when grown in media lacking SB202190 as compared to regular organoid media (Fig. 3a). Despite these findings, the absence of SB202190 did not significantly alter the secretion of chemokines by colonoids in response to FliC treatment, even though a trend for increase cytokine production was observed (Fig. 3c). We further assessed the effect of IL-37 on FliC stimulated colonoids grown without SB202190. Here we observed that SB202190 suppressed the full biological activity of IL-37 since its removal enabled IL-37 to decrease CCL20 secretion by 46% (Fig. 3d) as compared to the 25% reduction displayed with colonoids grown in regular organoid media (with SB202190) (Fig. 2b). Surprisingly, removal of SB202190 did not further decrease IL-37 mediated suppression of IL-8 release (Figs. 2b, 3d). Thus, aside from showing that the presence of SB202190 limits p38 activation in colonoids, we also confirm that IL-37 inhibits both p38 and NFκB activation and downstream signaling pathways, by showing a reduction in the phosphorylation of NFκB and p38, but with no modulation of total protein levels (Fig. 3e).

### Chemokine production by murine colonoids

**Figure 4:**
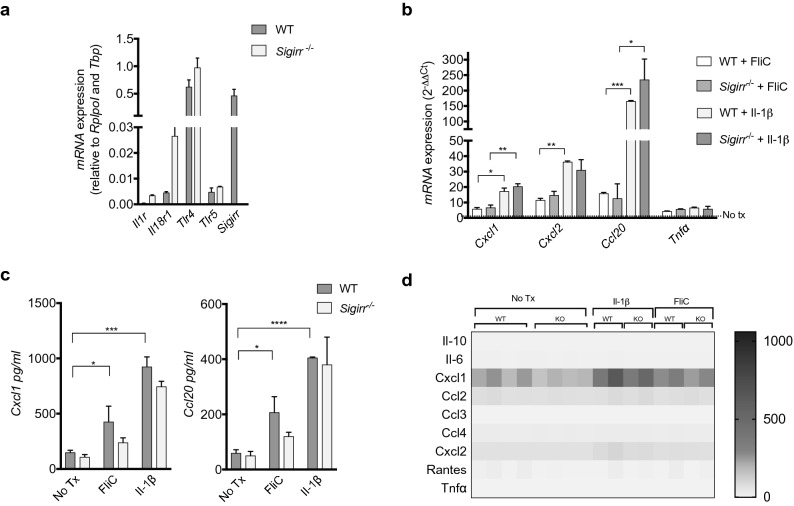
Mouse IEC responses to host (Il-1β) and bacteria stimuli (FliC). (**a**) qPCR analysis of innate receptor and negative regulator gene transcription by mouse colonoids expressed as relative expression over reference genes. (**b**) qPCR analysis of chemokines and cytokines after 4 h of stimulation with FliC and Il-1β expressed as fold change over untreated WT (*Sigirr*^+*/*+^) colonoid expression. (**c**) Cxcl1 (left) and Ccl20 (right) protein levels secreted basolaterally by colonoids derived from WT and *Sigirr*^−/−^ mice after 4 h of stimulation with FliC or Il-1β. (**d**) Heat map representation of various chemokine concentration in the supernatant of colonoids after 4 h of stimulation with FliC or Il-1β. Mean and SEM are indicated. Mean and SEM are indicated from n = 4 mouse organoid lines derived from each genotype. All data shown are representative of at least 2–3 independent experiments. Statistical significance calculated using one-way ANOVA *, *P* = 0.01 to 0.05; **, *P* = 0.001 to 0.01; ***, *P* = 0.0001 to 0.001; ***, *P* = 0.00001 to 0.0001. Comparison lacking annotations are not significant.

Currently, a murine homologue of IL-37 has not been identified, however previous studies have shown that human IL-37 can efficiently act on murine monocytes and lymphocytes to suppress innate immune signaling^14^. We examined whether IL-37 could also suppress immune signaling within murine colonoids, and whether such actions would depend on Sigirr expression. Colonic colonoids were generated from *Sigirr* deficient (*Sigirr*^−/−^) mice^13^, and their littermate controls (*Sigirr*^+/+^), and were grown in 3D culture as previously described^25, 26, 28^. We first determined the capacity of murine colonoids to respond to pro-inflammatory stimuli by evaluating the expression of various innate receptors. As shown in Fig. 4a, both *Sigirr*^+/+^ (WT) and *Sigirr*^−/−^ (KO) colonoids expressed higher levels of *Tlr4*, *Tlr5 Il1r* and *Il18r1* (Fig. 4a). Moreover, WT colonoids showed *Sigirr* gene expression whereas its total absence was observed in KO colonoids (Fig. 4a).

The murine colonoids were then stimulated with either *Salmonella* flagellin (FliC) (100 ng/ml), or murine Il-1β (10 ng/ml) to match the inflammatory stimuli used on human colonoids. Key murine inflammatory genes were then assessed including the chemokines *Ccl20*, *Cxcl2*, *Cxcl1* (murine homologue of IL-8) and the cytokine *Tnfα*. All genes showing significantly increased transcription upon stimulation, with Il-1β inducing the greatest increases (30 to 100-fold change) (Fig. 4b). ELISA analysis also showed significant amounts of Cxcl1 and Ccl20 secreted after FliC and Il-1β stimulation (Fig. 4c), with Il-1β being the more potent stimulus as opposed to FliC being stronger in human colonoids. To further characterize the innate response of these murine colonoids, we used a Milliplex Luminex assay covering 9 cytokines and chemokines (Il-6, Il-10, Cxcl1, Cxcl2, Ccl2, Ccl3, Ccl4, Ccl5, Tnfα). Surprisingly, the only secreted molecule detected on our panels was Cxcl1, whereas the other molecules (Il-6, Il-10, Cxcl2, Ccl2, Ccl3, Ccl4, Ccl5, Tnfα) were not significantly induced (Fig. 4d). These levels of Cxcl1 were similar to those detected by ELISA, confirming that this chemokine is strongly induced after FliC stimulation and was induced even more strongly following Il-1β stimulation (Fig. 4c).

### Sigirr mediates the effect of IL-37 on murine colonoids

**Figure 5:**
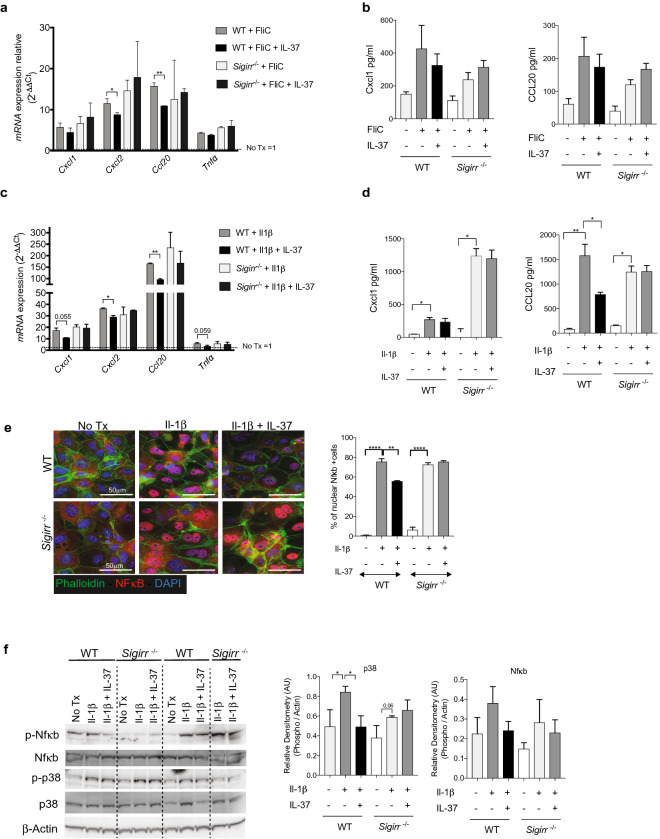
IL-37′s suppression of innate responses in mouse organoids is Sigirr dependent. (**a**) qPCR analysis of main inflammatory genes after FliC stimulation with IL-37 for 4 h on *Sigirr*^+/+ (^WT) and *Sigirr*^−/−^ mouse colonoids expressed as fold change over untreated colonoids. (**b**) Cxcl1 (left) and Ccl20 (right) protein levels secreted basolaterally by WT and *Sigirr*^−/−^ mouse colonoids after 4 h of stimulation with FliC and IL-37. (**c**) qPCR analysis of key inflammatory genes after with Il-1β stimulation, in concert with IL-37 for 4 h on *Sigirr*^+/+^ (WT) and *Sigirr*^−/−^ mouse colonoids expressed as fold change over untreated colonoids. (**d**) Cxcl1 (left) and Ccl20 (right) protein levels secreted basolaterally by WT and *Sigirr*^−/−^ mouse colonoids after 4 h of stimulation with Il-1β and IL-37. (**e**) Immunostaining against Nfkb (red), Actin Phalloidin (green) and DAPI (blue) of 2D monolayer after 30 min of stimulation with Il-1b with or without IL-37 (left). Counts of Nfkb positive nuclei from immunostaining (Right). (**f**) Western blot analysis of phospho and total NFkB, phospho and total p38 in mouse colonoids after 30 min of stimulation with Il-1β with or without IL-37 (left). Equal loading was confirmed with β-Actin as well as total protein stain of membrane (see Fig. S6 online). Densities relative to total protein or β-Actin are shown (right). Mean and SEM are indicated from n = 4 mouse organoid lines derived from each genotype. All data shown are representative of at least 3 independent experiments. Statistical significance calculated using one-way ANOVA *, *P* = 0.01 to 0.05; **, *P* = 0.001 to 0.01; ***, *P* = 0.0001 to 0.001; ***, *P* = 0.00001 to 0.0001. Comparison lacking annotations are not significant.

We next sought to clarify whether IL-37 would suppress this innate response of murine colonoids, as well as determine whether its actions depended on the expression of Sigirr. Upon addition of IL-37 at the same time as FliC, and measured after 4 h, IL-37 was found to successfully reduce inflammatory responses in *Sigirr*^+/+^ colonoids. IL-37 significantly reduced mRNA levels of *Cxcl1* (reduced 25%), *Cxcl2* (reduced 25%) and *Ccl20* (33%) as compared to colonoids not receiving IL-37, however this suppression was not observed with *Sigirr*^−/−^ colonoids (Fig. 5a). To clarify whether these IL-37 effects were seen at the protein level, we analyzed chemokine/cytokine secretion by ELISA. The same trends were observed at the protein level, with Cxcl1 and Ccl20 showing the greatest reductions in levels when IL-37 was added to stimulated *Sigirr*^+/+^ colonoids and this suppression was not observed with *Sigirr*^−/−^ colonoids (Fig. 5b).

Similar effects were observed when Il-1β stimulation was combined with IL-37 treatment. IL-37 reduced mRNA levels of *Cxcl1* (reduced 33%), *Ccl20* (reduced 43%) and to a lesser degree Tnfα, in *Sigirr*^+/+^ colonoids. This suppression was not observed in *Sigirr*^−/−^ colonoids suggesting that the suppressive effects of exogenous IL-37 on murine IEC is Sigirr dependent (Fig. 5c). These IL-37 effects were also observed at the protein level, with Cxcl1 and Ccl20 secretion significantly reduced when IL-37 was added to stimulated *Sigirr*^+/+^ colonoids (Fig. 5d). This suppression of innate signaling was not observed in *Sigirr*^−/−^ colonoids following treatment with IL-37 (Fig. 5d). These data suggest that the ability of IL-37 to suppress inflammatory signaling within murine IEC is dependent on the expression of Sigirr.

Due to our earlier results that demonstrate IL37 inhibits Nfκb activation in human derived colonoids and Nfκb is downstream of Sigirr signaling pathways, we explored if IL37 exerts its inhibitory effect on Nfκb through its use of Sigirr^30–32^. We again utilized our 2D colonoid-derived monolayer system and assessed Nfκb nuclear translocation and activation through immunostaining. Monolayers derived from *Sigirr*^+/+^ and *Sigirr*^−/−^ mouse colonoids were treated with inflammatory stimuli (Il-1β (10 ng/ml) or FliC (100 ng/ml)) for 30 min to induce Nfκb translocation to the nucleus (Fig. 5e for Il-1β) (See Fig. S3 online for FliC). When the monolayers of either genotype were stimulated with Il-1β, the majority of cells showed nuclear co-localisation of Nfκb (red) and DAPI (blue) (Fig. 5e). When the stimulated monolayers were treated with IL-37, there was a significant reduction in the number of positive nuclear Nfκb cells in the *Sigirr*^+/+^ colonoid monolayers that was not observed in the *Sigirr*^−/−^ monolayers (Fig. 5e). A similar reduction in nuclear Nfκb staining was observed with *Sigirr*^+/+^ colonoids, but not with *Sigirr*^−/−^ colonoids, when both were treated with FliC plus IL-37 (see Fig. S3 online). Similar to our findings with human organoids, Western blot analysis on mouse colonoids confirmed that IL-37 reduces both Nfκb activation and p38 after Il-1β stimulation (Fig. 5f). The same trend was also observed for FliC-treated colonoids in the presence of IL-37 (data not shown). These results suggest that extracellular IL-37 acts on IEC through a Sigirr dependent pathway to inhibit FliC or Il-1β induced Nfκb and p38 activation.

## Discussion

IL-37 is a novel anti-inflammatory cytokine that plays an important role in regulating innate immunity. The majority of studies on IL-37 have focused on its ability to regulate the function of immune cells in various tissues and diseases, whereas its potential effects on the gut epithelium have been largely overlooked^19, 20^. By using human and murine colonoids and colonoid-derived monolayers, we show for the first time that extracellular IL-37 can effectively suppress IEC inflammatory responses in a manner dependent on Sigirr (in murine colonoids). Specifically, IL-37 reduced NFκB and p38 activation and chemokine secretion typically seen after stimulation of colonoids with bacterial FliC, as well as the cytokine Il-1β. This is the first study showing that IL-37 can suppress immune signaling in human and murine colonoids and that the effect is Sigirr dependent in mice.

Through the use of human colonoids, we investigated their innate immune responses and the effects of IL-37 on primary tissue-derived IEC, that unlike cell lines, have not undergone transformation or cell culture adaptation. Organoids provide several advantages over traditional cell culture methods, since they recapitulate the physiological and biological properties of the tissue of origin. Our results highlight the inter-individual variability that can occur when studying primary tissues and organoids derived from patients. Each colonoid line likely behaves in ways influenced by the specific genetic, epigenetic and environmental factors that influenced the original donor. Our results also surprisingly reveal that human colonoids are unresponsive to IL-1β stimulation despite expressing *IL1R*, as compared to the transformed Caco-2 cell line. In contrast, murine derived colonoids were found to respond very actively to the Il-1β stimulus, emphasizing previous findings that species differences can greatly affect IEC behavior and their innate immune responses^3, 27^.

Interestingly, our results also revealed that even picomolar concentrations (1000 pg/ml to 100 pg/ml) of IL-37 are sufficient to significantly inhibit murine and human IEC innate immune responses with micromolar concentration being less effective. The finding that higher doses of IL-37 can be less effective has been previously described in human macrophages, yet the mechanisms have yet to be clearly defined^21^. Our results also support previous findings that IL-37 can suppress the secretion of several chemokines such as CCL2, CCL5 and CXCL8 by the transformed T84 IEC line^33^. While this earlier study explored the ability of T84 cells to express IL-37, our results suggest that non-transformed colonic IEC do not produce significant levels of IL-37. Our experimental design instead used recombinant IL-37, enabling our assessment of extracellular IL-37 actions in its secreted form. This approach also gives us the advantage of discriminating the extracellular effect of IL-37 on IEC over the effect it could potentially have when produced intracellularly, as demonstrated with the use of CRISPR/Cas9 to delete IL37 in T84 cells.

In this study, IL-37 suppressed chemokine secretion levels from colonoids by 20 to 45%. We believe this would be biologically significant, and in keeping with previous reports, that IL-37 does not fully inhibit innate immune response but rather serves to balance excessive inflammation. It would be interesting to verify is this chemokine inhibition would translate into more physiological effects such as reduced chemotaxis of immune cells, normally induced by these chemokines. Unfortunately, using the current organoid system would be impractical since the chemokine levels are too low to induce migration of neutrophils or other immune cells. A scale-up of the organoid cultures (30–50 fold) or a co-culture system could help to answer these questions in the future.

In cells that produce IL-37, IL-37 can act intracellularly by binding to SMAD3 and then translocate to the nucleus to repress transcription of specific target genes^14, 22, 34^. IL-37 can also be secreted into the extracellular milieu where it will bind to specific heteroreceptors (IL-18 and SIGIRR) to inhibit innate immune signaling within its target cells^21, 24^. By using recombinant IL-37, we focused our efforts on defining the role of extracellular IL-37 on IECs. While we cannot rule out the possibility that some effects seen in human colonoids could reflect the actions of intrinsic IL-37 production, we found that human colonoids in fact produce very low levels of IL-37, as assessed by both qPCR and ELISA. Nevertheless, it is possible that in vivo, and particularly during disease states, IEC may produce greater levels of IL-37, since some studies have shown strong immunohistochemical staining for IL-37 in the inflamed gut epithelium of IBD patients^22^. In our system, we detected low levels of IL-37 mRNA without seeing any increase in protein expression (see Fig. S1 online). This suggests that colonic IEC from healthy individuals might be missing key elements (transcription factor, cytokine stimulation, differentiation state) required to produce functional levels of IL-37. Further investigations should focus on IL-37 expression by IECs and under which conditions, and by which IEC subtypes it may be expressed.

IL-37 does not appear to be expressed by mice or its human orthologue has not been discovered yet^35^. However previous studies have shown that human IL-37 can act on murine cells in a similar fashion to its actions on human cells. By using murine colonoids, we confirmed that human recombinant IL-37 is effective on murine IECs, providing a new system to study IL-37 and it signaling pathways and downstream targets. In this study, colonoids derived from *Sigirr*^+/+^ and *Sigirr*^−/−^ mice were used to prove that extracellular IL-37 requires Sigirr expression to suppress inflammatory signaling. This data suggests that IL-37 acts on mouse IEC in a similar manner to what has been previously published with immune cells^21^. SIGIRR’s ability to regulate gut inflammation and innate immune responses by IEC have been previously described in different in vitro and in vivo models^9, 12, 13^. Here, our data support the concept that Sigirr, along with the IL-18R, functions as a hetero-receptor that mediates the regulator actions of IL-37. While the exact mechanisms remain to be determined, it is tempting to speculate that during clinical forms of intestinal inflammation, IL-37 could be secreted in the environment to act on SIGIRR expressing IEC to repress innate immune signaling, limiting the development of excessive inflammation. These speculations certainly require further clarification. However, our study is the first study to support the hypothesis that IL37 can act on primary IEC and further studies are needed to better understand how IL-37 and SIGIRR interact to balance inflammatory responses in the gut.

In summary, our study has demonstrated important differences in the innate immune response of human and murine primary IEC. While human IEC are highly reactive to FliC stimulation, mouse IEC produce stronger innate immune responses following Il-1β stimulation than they do when exposed to FliC. Moreover, these species differences are reflected in the panel of chemokines induced, with human IEC secreting a greater array of immune molecules (CCL2, CCL5, CCL20, IL8). By taking advantage of the colonoid system, we show for the first time, the anti-inflammatory role of IL-37 on primary human IEC by reducing NFκB and p38 signalling while we corroborated that its extracellular role depends on Sigirr expression in mouse IEC. Taken together, these data show that both human and mouse colonoids respond (albeit differently) to bacterial products, and their responses can be suppressed by IL-37, through its ability to signal through SIGIRR.

## Methods

### Ethics statement

De-identified intestinal biopsies from paediatric and adult patients as well as blood from adult patients were collected using experimental methods and protocols approved by the Clinical Research Ethics Boards of the University of British Columbia, the British Columbia Children’s and Women’s Research Review Committee, and from the Providence Health and Clinical Research Ethics Board (H14-0391, H15-01977, H07-02861). Written informed consent was obtained from all participants (or legal guardians) prior to inclusion in this study. All following investigations and methods were performed in accordance with relevant guidelines and regulations of the Tri-council Policy Statement: Ethical Conduct for Research Involving Humans (Government of Canada) and followed the principles of the Declaration of Helsinki.

All mouse experiments were performed according to protocols approved by the University of British Columbia's Animal Care Committee and in direct accordance with the Canadian Council on Animal Care (CCAC). Authors complied to ARRIVE guidelines for reporting animal research.

### Mouse strain

*Sigirr*^*−/−*^ mice have been described previously^10^. *Sigirr*^*−/−*^ and *Sigirr*^+*/*+^ mice were bred under specific pathogen-free conditions at the BC Children’s Hospital Research Institute, with male mice (4 mice total from each genotype) being used for organoids isolation at 8–12 weeks of age.

### Generation of mouse colonoids and maintenance

Murine colonoids were isolated as previously described. In brief, mouse colonic crypts were isolated and washed before being diluted 1:1 in Matrigel (Corning). After the Matrigel solidified, organoid media (base media (Advanced DMEM/F12, supplemented with Pen Strep GlutaMAX and HEPES) with 50% WRN supplemented with N2 (Invitrogen), B27 (Invitrogen), N-acetylcystine (Sigma-Aldrich), nicotinamide (Sigma-Aldrich), mEGF (Invitrogen), A83-01 (Tocris), SB 202,190 (Sigma-Aldrich), Y-27632 (AbMole)) was added to the well and incubated at 37 °C with 5% CO_2_. Media was changed every three days and the colonoids were passaged every five to seven days.

### Generation of human colonic colonoids from biopsies and maintenance

Intestinal crypts were isolated from de-identified pediatric or adult sigmoid colon biopsies and grown as colonoid cultures using well described methods^26, 28, 36^. The solution containing the colonic crypts was centrifuged and washed twice with base media then diluted 1:1 in Matrigel (Corning). This was pipetted into multiple domes in a 24-well plate and incubated at 37 °C with 5% CO_2_. After the Matrigel solidified, organoid media (same as mouse organoids) was added to the well and incubated at 37 °C with 5% CO_2_. Media was changed every three days and the colonoids were passaged every seven to ten days.

### Stimulation of human and mice colonoids

For experiments, colonoids were passaged using TrypLE techniques as already described but with modifications^3, 27, 29^. First, Matrigel domes were disrupted by adding ice-cold PBS containing 5 mM EDTA and incubating on ice for 15 min. Colonoids were washed twice with base media, resuspended in TrypLE express (Gibco) and incubated at 37 °C with 5% CO_2_ for 2 × 5 min. The colonoids were then rapidly disrupted into single cell suspensions with repeated pipetting through a p1000 tip, and an equal volume of organoid media was added. Cells were centrifuged, then resuspended in base media. The cells were counted using an automated cell counter and the appropriate number of cells (15,000 cells/well for human; 10,000 cells/well for mouse) were seeded using Matrigel as described in the colonoid isolation section in a 24-well plate. Colonoids were stimulated (days 5–7 after seeding for mice, day 10–14 for human) with base media with FliC (100 ng/ml; InvivoGen) or human IL-1β (10 ng/mL; Sigma) or murine IL-1β (10 ng/mL; PeproTech) or LPS O55:B5 (1000 ng/ml; InvivoGen) with or without recombinant IL-37 (100 to 0,1 ng/ml; R&D) or corresponding volume of base media for 4 h.

### Colonoid-derived monolayer seeding and stimulation

Monolayers derived from colonoids (human or mice) were generated as previously described with modifications^27–29^. First, the growth media was removed, then Matrigel domes were disrupted and colonoids were resuspended in TrypLE express (Gibco) and incubated at 37 °C with 5% CO_2_ for 2 × 5 min. The colonoids were then rapidly disrupted into single cell suspensions with gentle pipetting through a p1000 tip, and an equal volume of monolayer media (base media supplemented with 50% WRN, N2 (Invitrogen), B27 (Invitrogen), mEGF (Invitrogen) and Y-27632 (AbMole) was added. Cells were centrifuged, then resuspended in monolayer media and added dropwise to Geltrex (Gibco) coated coverslips in 24-well plates. Monolayers were incubated at 37 °C with 5% CO_2_ and media changed 24 h after seeding. 72 h after seeding, confluent monolayers were stimulated with monolayer media supplemented with FliC (100 ng/ml; InvivoGen) or *m*IL-1β (10 ng/mL; Invitrogen) with or without recombinant IL-37 (100 ng/ml; R&D systems) or a corresponding volume of base media for 30 min.

### Monolayer immunostaining

For colonoid monolayer immunostaining, after 30 min of stimulation, coverslips were fixed in 4% PFA for 15 min at room temperature. Then the cells were rinsed in PBS 1X twice, and treated with PBS, 0.1% Triton X-100 and 0.05% Tween 20 for 15 min, then blocked with 2% donkey serum in PBS, 0.01% Triton X-100 and 0.05% Tween 20 overnight. Coverslips were then stained with anti-NFκB antibody (1:100; Cell Signaling #8242) overnight at 4 °C followed by secondary antibody staining with anti-rabbit Alex Fluor-4588 and Alexa Fluor 468-phalloidin for 60 min, washed and mounted using ProLong Gold Antifade reagent containing 4′,6-diamidino-2-phenylindole (DAPI) for DNA staining. Sections were viewed on a Zeiss AxioImager microscope and images taken using an AxioCam HRm camera operating through Zen software.

### Isolation of human peripheral blood mononuclear cells (PBMC)

Adult peripheral blood was collected in sodium heparin anti-coagulated Vacutainers (BD, Canada) from healthy adults (range 20 to 40 years old). Peripheral Blood mononuclear cells (PBMC) were isolated from de-identified adult healthy donors as it is previously described^37, 38^. In brief, PBMC were isolated through density centrifugation using LymphoPrep (STEMCELL technologies) and resuspended RPMI1640 (Roswell Park Memorial Institute medium) supplemented with 2 mM L-glutamine (Gibco), 10% fetal bovine serum,10% and Pen-strep (Gibco), and 1% *Penicillin–Streptomycin* referred to as R10 medium.

### LPS stimulation of human PBMCs

PBMC were treated as previously described^23, 38^. Briefly, PBMC were cultured in R10 medium as it was described above. PMBC (0.5 × 10^6^ per well) were stimulated in a round-bottomed 96 well-plate and stimulated with LPS (100 ng/ml; InvivoGen). After 24 h of stimulation, cells were pelleted and the supernatant was collected for IL-37 analysis by ELISA.

### RNA extraction for colonoids

For mRNA extraction after simulation, Matrigel was disrupted as described in previous sections. Harvested colonoids were resuspended in 300 ul of Trizol and lysed at room temperature for 15 min. RNA was extracted using Direct-zol RNA micro-Prep (Zymo research) following the manufacturer’s protocol.

### Real time qPCR analysis

Total RNA from colonoids quantified using a NanoDrop 1000 Spectrophotometer (Thermo Fisher Scientific). 500 ng of RNA was reverse-transcribed using 5X All-In-One RT (abm), followed by dilution of the cDNA at 1:5 in RNase/DNasefree H_2_O. The qPCR was carried out using a Bio-Rad CFX connect Real-time PCR detection system, with the specificity for each of the PCR reactions confirmed by melting point analysis. Quantitation was performed using CFX Maestro software (Bio-Rad). All genes were normalized to two housekeeping genes. *Protein Lateral Stalk Subunit (Rplp0)* and *Glyceraldehyde-2-Phosphate Dehydrogenase (Gapdh)* were used as reference genes for all mouse qPCR experiments*; TATA-box binding protein (TBP)* and *Glyceraldehyde-2-Phosphate Dehydrogenase GAPDH* were used as reference genes for all human qPCR experiments. For Figs. 4a, S1B-C-D, *mRNA* transcript expression was normalized to the relative expression of the reference genes using the 2^-(ΔCt)^. For treatment conditions in Figs. 1a, 2a and 4b, *mRNA* transcript expression was normalized to the control group (untreated condition) using the 2^−(ΔΔCt)^ methods.

Primer sequences for all genes analyzed are summarized in Table 1.

**Table 1:** Primer list for qPCR analysis.

Target gene	Primer FWD	Primer REV
**Human primers**		
CCL20	GCT ACT CCA CCT CTG CGG CG	ACC TCC AAC CCC AGC AAG GTT CT
CXCL2	GAAAGCTTGTCTCAACCCCG	TGGTCAGTTGGATTTGCCATTTT
IL1R	ATGAAATTGATGTTCGTCCCTGT	ACCACGCAATAGTAATGTCCTG
IL18R1	AAGAACGCCGAGTTTGAAGAT	GAGCAGTTGAGCCTTACGTTT
IL37	AAGTCATCCATCCCTTCAGC	CCCACCTGAGCCCTATAAAA
IL8 (CXCL8)	TGT GTG AAG GTG CAG TTT TGC	GCA CCC AGT TTT CCT TGG GG
SIGIRR	GCTGACTGCAAGGACAGAGA	ACTCGTGGAGGCTGTAGTGG
TLR4	AAGCCGAAAGGTGATTGTTG	CTGAGCAGGGTCTTCTCCAC
TLR5	TGC ATC CAG ATG CTT TTC AG	TGC TGA TGG CAT TGC TAA AG
TNFa	TTCCAGAAGATGATCTGATGC	TCAGCCTCTTCTCCTTCCT
GAPDH (reference)	ATG ACC TTG CCA CAG CC	CCC ATC ACC ATC TTC CAG
TBP (reference)	GCC CGA AAC GCC GAA TAT	CCG TGG TTC GTG GCT CTC T
**Mouse primers**		
Cxcl1	TGC ACC CAA ACC GAA GTC AT	TTG TCA GAA GCC AGC GTT CAA
Cxcl2	CCT GCC AAG GGT TGA CTT CA	TTC TGT CTG GGC GCA GTG
Ccl20	GCC TCT CGT ACA TAC AGA CGC	CCA GTT CTG CTT TGG ATC AGC
Il1r1	AAGTAATGCTGTCCTGGGCT	AGCACTTTCATATTCTCCATTTGT
Tlr4	TGGCTGGTTTACACATCCATCGGT	TGGCACCATTGAAGCTGAGGTCTA
Tlr5	TGGAGCCGAGTGAGAAATCAG	GTGACGATCCTGGGGTGTTT
Sigirr	CCC TGC TCT ATG TTA AGT GTC G	TCA GGT TCA CCA AAA GGT CG
Tnfa	CATCTTCTCAAAATTCGAGTGACAA	TGG GAG TAG ACA AGG TAC AAC CC
Rplp0I (reference)	AGA TTC GGG ATA TGC TGT TGG C	TCG GGT CCT AGA CCA GTG TTC
Gapdh (reference)	ATG ACC TTG CCA CAG CC	CCC ATC ACC ATC TTC CAG

### Enzyme-linked immunosorbent assay (ELISA)

After 4 h stimulation of colonoids in Matrigel dome, the media was collected and centrifuged at 1000 g at 4 °C for 10 min, then the supernatant was collected and stored at − 80 °C. 100 uL of the supernatant (or dilution) was used per well in duplicate and the ELISAs were performed according to manufacturer’s instructions (human CCL20 (R&D systems), human IL-8 ELISA MAX Deluxe Set (BioLegend), murine Ccl20 and Cxcl1 (R&D systems)).

### Western blotting

Colonoids were resuspended in RIPA buffer with protease inhibitors and phosphatase inhibitor, sonicated, then centrifuged at 16,000xg for 20 min at 4 °C. Total protein was estimated and 10 μg of whole cell lysate prepared according to manufacturer’s instructions in 1X Bolt LDS Sample Buffer with 1X Bolt Reducing Agent (Life Technologies) and heated at 70 °C for 10 min. Proteins were separated by Bolt 12% Bis–Tris Gel (Life Technologies), transferred to PVDF membrane (Life Technologies), followed by immunoblotting with rabbit monoclonal phospho-p38 (1:2000; Cell Signaling Technologies #9211), total p38 (1:2000; Cell Signaling Technologies #9212), phospho-NFκB (1:2000; Cell Signaling Technologies #3033), total NFκB (1:2000; Cell Signaling Technologies #8242) or mouse monoclonal anti-β-actin (1:2000; ABM # G043), then with horse α-rabbit IgG:HRP (1:2000; Cell Signaling Technologies #7076) or horse α-mouse IgG:HRP (1:2000; Cell Signaling Technologies #7076). Western blot images were taken using a ChemiDoc imaging system (Bio-Rad) and densitometry analysis of the obtained images were done using ImageJ 1.45S software (Wayne Rasband, NIH).

### Milliplex luminex assay

Human colonoid supernatant (25 μl) was used to measure cytokine concentrations using a custom-designed multi-analyte Cytokine Human Magnetic Panel bead array, (MilliporeSigma) consisting of CCL2 (MCP-1), CCL3 (MIP-1α), CCL5 (Rantes), IL-10, IL-8, IL-18, IL-6, and TNF*α,* according to the manufacturer’s protocol. Mouse colonoid supernatant (25 μl) was used on a custom-designed multi-analyte Cytokine Mouse Magnetic Panel bead array, (Millipore, Sigma) consisting of Ccl2 (Mcp-1), Ccl3 (Mip-1α), Ccl4 (Mip-1β) Ccl5 (Rantes), Il-10, Il-8, Il-18, Il-6, and Tnfα, according to the manufacturer’s protocol*.* Results were obtained with a Flexmap 3D system with Luminex xPONENT software version 4.2 (both from Luminex Corp.; Austin, TX, USA). Cytokine concentrations were determined using Milliplex Analyst software (version 3.5.5.0, Millipore).

### Statistical analysis

All results presented in this study are expressed as the mean values ± standard errors (SEM). Mann–Whitney U-test, student *t*-test and one-way ANOVA were performed using GraphPad Prism software, version 7.0c for MAC OS X. A *p* value of 0.05 or less was considered significant, with asterisks denoting significance in the figures.

## Supplementary Information

Supplementary Information.

## Data Availability

All data and reagents are available upon request to the corresponding author. No data sets were generated during the current study.

## References

[1] Allaire JM (2018). The intestinal epithelium: central coordinator of mucosal immunity. Trends Immunol..

[2] Price AE (2018). A map of toll-like receptor expression in the intestinal epithelium reveals distinct spatial, cell type-specific, and temporal patterns. Immunity.

[3] Kayisoglu O (2020). Location-specific cell identity rather than exposure to GI microbiota defines many innate immune signalling cascades in the gut epithelium. Gut.

[4] Abreu MT (2010). Toll-like receptor signalling in the intestinal epithelium: How bacterial recognition shapes intestinal function. Nat. Rev. Immunol..

[5] Frantz AL (2012). Targeted deletion of MyD88 in intestinal epithelial cells results in compromised antibacterial immunity associated with downregulation of polymeric immunoglobulin receptor, mucin-2, and antibacterial peptides. Mucosal Immunol..

[6] Gibson DL (2008). MyD88 signalling plays a critical role in host defence by controlling pathogen burden and promoting epithelial cell homeostasis during Citrobacterrodentium-induced colitis. Cell Microbiol..

[7] Ruan W (2020). Enhancing responsiveness of human jejunal enteroids to host and microbial stimuli. J. Physiol..

[8] Kadota C (2010). Down-regulation of single immunoglobulin interleukin-1R-related molecule (SIGIRR)/TIR8 expression in intestinal epithelial cells during inflammation. Clin. Exp. Immunol..

[9] Khan MA (2010). The single IgG IL-1–related receptor controls TLR responses in differentiated human intestinal epithelial cells. J. Immunol..

[10] Wald D (2003). SIGIRR, a negative regulator of Toll-like receptor - Interleukin 1 receptor signaling. Nat. Immunol..

[11] Guo J, Zhan X, Xu G, Mao C, Wei R (2020). Transcriptomic analysis reveals that IL-1R8/Sigirr is a novel macrophage migration regulator and suppresses macrophage proliferation through p38 MAPK signaling pathway. Biomed. Pharmacother..

[12] Sham HP (2013). SIGIRR, a negative regulator of TLR/IL-1R signalling promotes microbiota dependent resistance to colonization by enteric bacterial pathogens. PLoS Pathog..

[13] Xiao H (2007). The Toll-interleukin-1 receptor member SIGIRR regulates colonic epithelial homeostasis, inflammation, and tumorigenesis. Immunity.

[14] Nold MF (2010). IL-37 is a fundamental inhibitor of innate immunity. Nat. Immunol..

[15] Huang N (2018). Interleukin-37 alleviates airway inflammation and remodeling in asthma via inhibiting the activation of NF-κB and STAT3 signalings. Int. Immunopharmacol..

[16] Imaeda H (2013). Epithelial expression of interleukin-37b in inflammatory bowel disease. Clin. Exp. Immunol..

[17] Wang YC, Weng GP, Liu JP, Li L, Cheng QH (2019). Elevated serum IL-37 concentrations in patients with sepsis. Medicine (Baltimore).

[18] Jiang M (2018). IL-37 inhibits invasion and metastasis in non-small cell lung cancer by suppressing the IL-6/STAT3 signaling pathway. Thorac. Cancer.

[19] Theoharides TC, Tsilioni I, Conti P (2019). Mast cells may regulate the anti-inflammatory activity of IL-37. Int. J. Mol. Sci..

[20] Wang DW (2016). Interleukin-37 enhances the suppressive activity of naturally occurring CD4+ CD25+ regulatory T cells. Sci. Rep..

[21] Li S (2015). Extracellular forms of IL-37 inhibit innate inflammation in vitro and in vivo but require the IL-1 family decoy receptor IL-1R8. Proc. Natl. Acad. Sci. U. S. A..

[22] Bulau AM (2014). Role of caspase-1 in nuclear translocation of IL-37, release of the cytokine, and IL-37 inhibition of innate immune responses. Proc. Natl. Acad. Sci. U. S. A..

[23] Rudloff I (2017). Monocytes and dendritic cells are the primary sources of interleukin 37 in human immune cells. J. Leukoc. Biol..

[24] Nold-Petry CA (2015). IL-37 requires the receptors IL-18Rα and IL-1R8 (SIGIRR) to carry out its multifaceted anti-inflammatory program upon innate signal transduction. Nat. Immunol..

[25] Sato T (2009). Single Lgr5 stem cells build crypt-villus structures in vitro without a mesenchymal niche. Nature.

[26] Sato T (2011). Long-term expansion of epithelial organoids from human colon, adenoma, adenocarcinoma, and Barrett’s epithelium. Gastroenterology.

[27] Holly MK (2020). Salmonella enterica infection of murine and human enteroid-derived monolayers elicits differential activation of epithelium-intrinsic inflammasomes. Infect. Immun..

[28] Fernando EH (2017). A simple, cost-effective method for generating murine colonic 3D enteroids and 2D monolayers for studies of primary epithelial cell function. Am. J. Physiol. Gastrointest. Liver Physiol..

[29] Crowley SM (2020). Intestinal restriction of Salmonella Typhimurium requires caspase-1 and caspase-11 epithelial intrinsic inflammasomes. PLoS Pathog..

[30] Riva F (2012). TIR8/SIGIRR is an interleukin-1 receptor/toll like receptor Family member with regulatory functions in inflammation and immunity. Front. Immunol..

[31] Garlanda C (2004). Intestinal inflammation in mice deficient in Tir8, an inhibitory member of the IL-1 receptor family. Proc. Natl. Acad. Sci. U. S. A..

[32] Garlanda C, Riva F, Bonavita E, Gentile S, Mantovani A (2013). Decoys and regulatory ‘receptors’ of the il-1/toll-like receptor superfamily. Front. Immunol..

[33] Günaltay S, Ghiboub M, Hultgren O, Hörnquist EH (2017). Reduced IL-37 production increases spontaneous chemokine expressions in colon epithelial cells. Dig. Dis. Sci..

[34] Sharma S (2008). The IL-1 family member 7b translocates to the nucleus and down-regulates proinflammatory cytokines. J. Immunol..

[35] Cavalli G, Dinarello CA (2018). Suppression of inflammation and acquired immunity by IL-37. Immunol. Rev..

[36] Rees WD (2020). Enteroids derived from inflammatory bowel disease patients display dysregulated endoplasmic reticulum stress pathways, leading to differential inflammatory responses and dendritic cell maturation. J. Crohns. Colitis.

[37] Kan B (2018). Cellular metabolism constrains innate immune responses in early human ontogeny. Nat. Commun..

[38] de Goede OM, Lavoie PM, Robinson WP (2017). Cord blood hematopoietic cells from preterm infants display altered DNA methylation patterns. Clin. Epigenetics.

