# Supplementary material for: Interleukin-37 regulates innate immune signaling in human and mouse colonic organoids
Source: Sci Rep. 2021 Apr 15;11:8206. doi: 10.1038/s41598-021-87592-2 (PMC8050237; doi:10.1038/s41598-021-87592-2)
Supplement: Supplementary file 1 — Supplementary Information. [file 41598_2021_87592_MOESM1_ESM.pdf]

Interleukin-37 regulates innate immune signaling in human and mouse colonic organoids

Joannie M Allaire[1], Anita Poon[1], Shauna M Crowley[1], Xiao Han[1], Zohreh Sharafian[2], Navjit Moore[1], Martin Stahl[1,3] Brian Bressler[4], Pascal M Lavoie[2], Kevan Jacobson[1], Xiaoxia Li[5], Bruce A Vallance[1*].

[1]Department of Pediatrics, BC Children's Hospital, University of British Columbia, Vancouver, British Columbia, Canada.
[2]Division of Neonatology, Department of Pediatrics, BC Children's Hospital, University of British Columbia, Vancouver, BC, Canada.
[3]STEMCELL Technologies, Vancouver, British Columbia, Canada.
[4]Division of Gastroenterology, Department of Medicine, University of British Columbia, Vancouver, BC, Canada.
[5]Department of Immunology, Cleveland Clinic Foundation, Cleveland, Ohio, USA.

*Corresponding author: Bruce A. Vallance (BAV) bvallance@cw.bc.ca

Figure S1

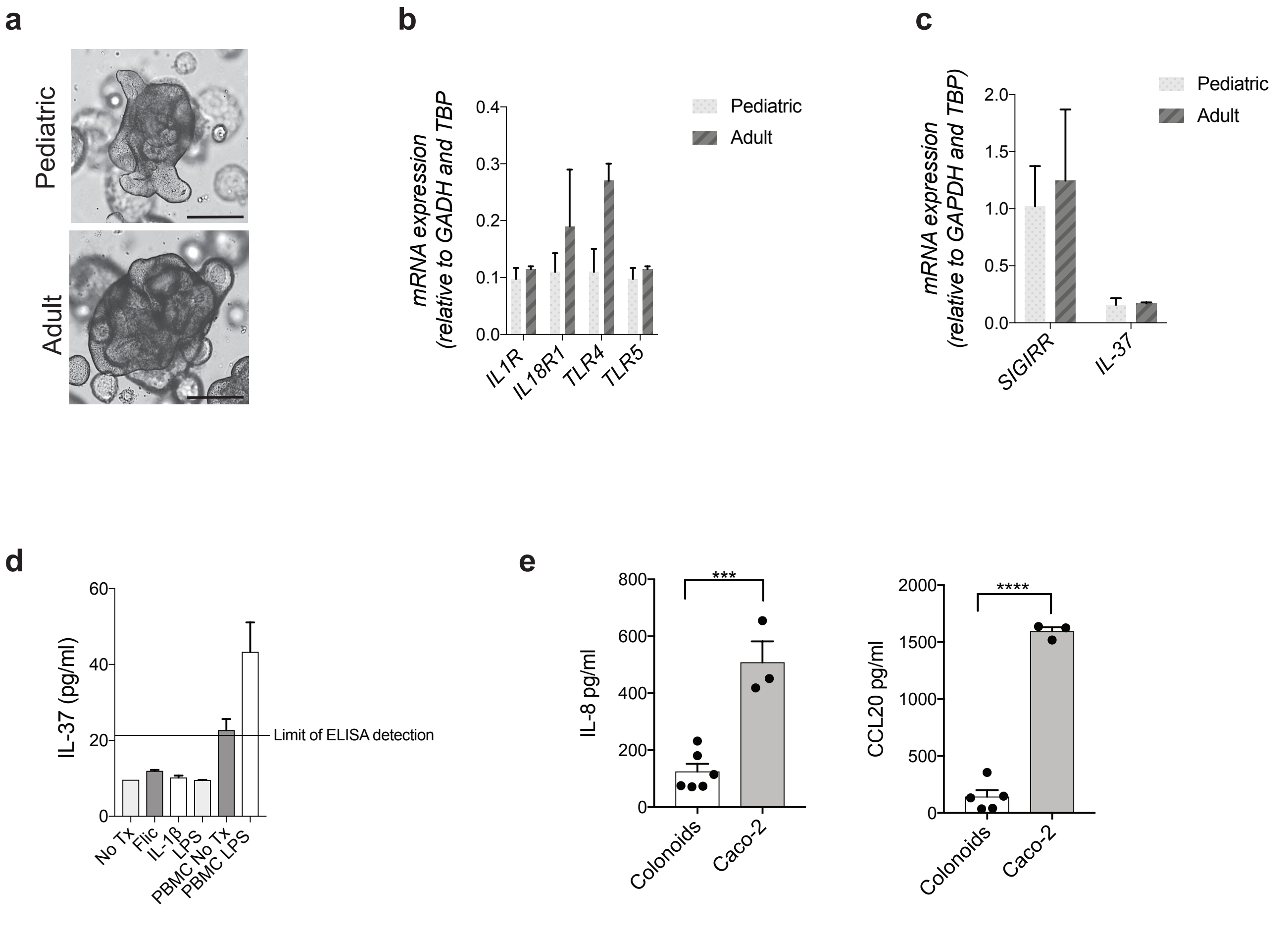

Figure S1: Innate receptor expression and IL-1β responses of human IEC
a) Brightfield images of representative colonoids derived from sigmoid colon biopsies taken from pediatric (top) and adult (bottom) donors. **b)** qPCR analysis of innate receptor expression by human colonoids as relative expression over reference genes. **c)** qPCR analysis of SIGIRR and IL-37 by human colonoids expressed as relative expression over reference genes. **d)** IL-37 levels secreted by human colonoids stimulated by FliC, IL1β and LPS for 24h versus PBMCs stimulated by LPS for 24h as a positive control. **e)** IL-8 and CCL20 protein levels secreted by human colonoids versus Caco-2 cell lines after 4h of stimulation with IL-1β. Mean and SEM are indicated from n= 6-8 donors (4 adult and 2-4 pediatric). All data shown are representative of at least 3 independent experiments.

Figure S2

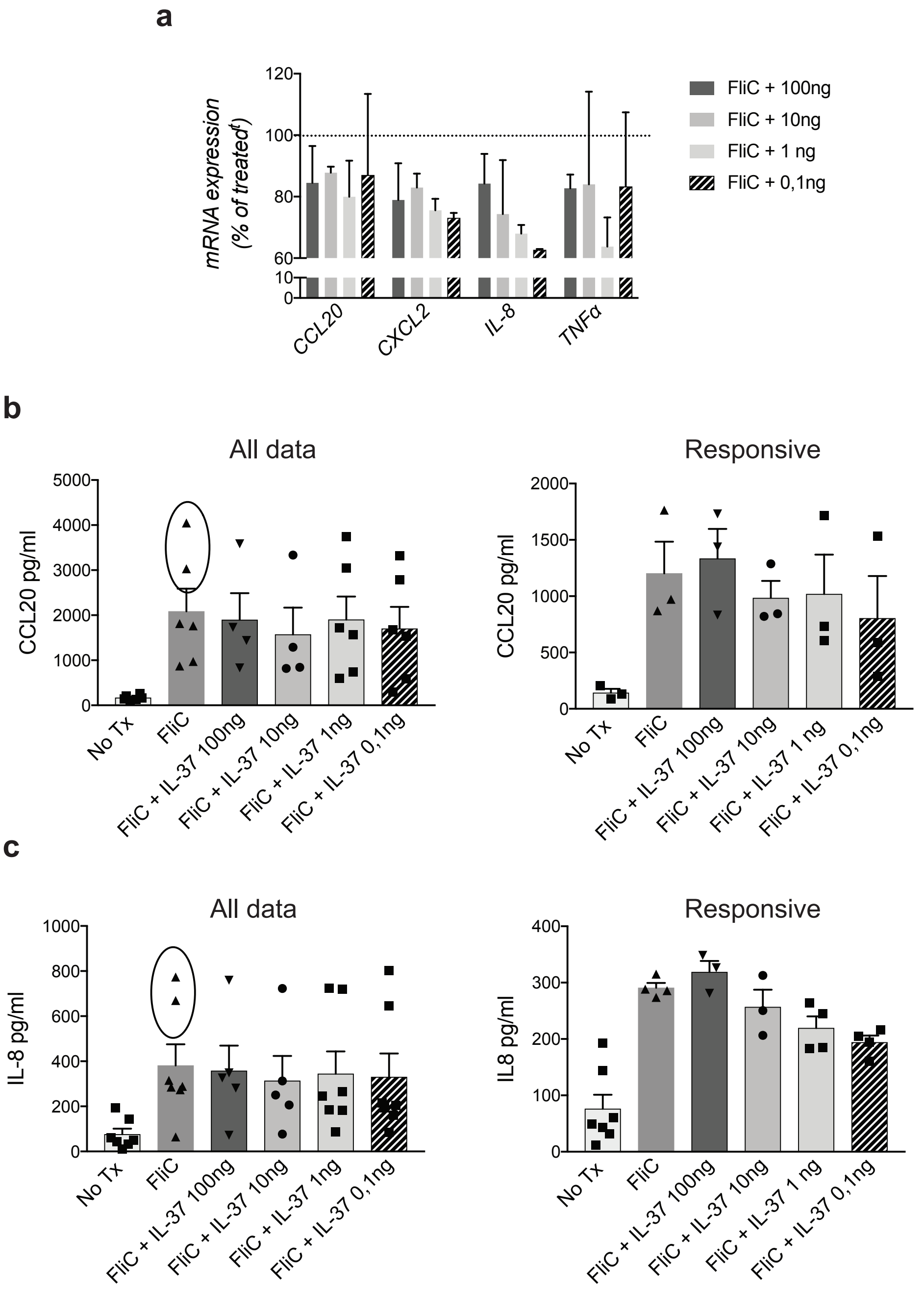

Figure S2: Dose response of IL-37 on human IEC responses to FliC stimulation.
a) qPCR analysis of inflammatory gene transcription after exposure to FliC for 4h and a gradient concentration of IL-37. **b)** CCL20 protein levels secreted basolaterally by human colonoids after 4h of stimulation simultaneously with various IL-37 doses, showing all data (left) and responsive donors only (right). **c)** IL-8 protein levels secreted basolaterally by human colonoids after 4h of simultaneous stimulation with FliC and different IL-37 doses, showing all data (left) and only responsive donors (right). Mean and SEM are indicated from n= 6 (4 adult, 2 pediatric) donors for all data, n= 3-4 (3 adult, 1 pediatric) donors for dose response experiments. All data shown are representative of at least 3 independent experiments.

Figure S3

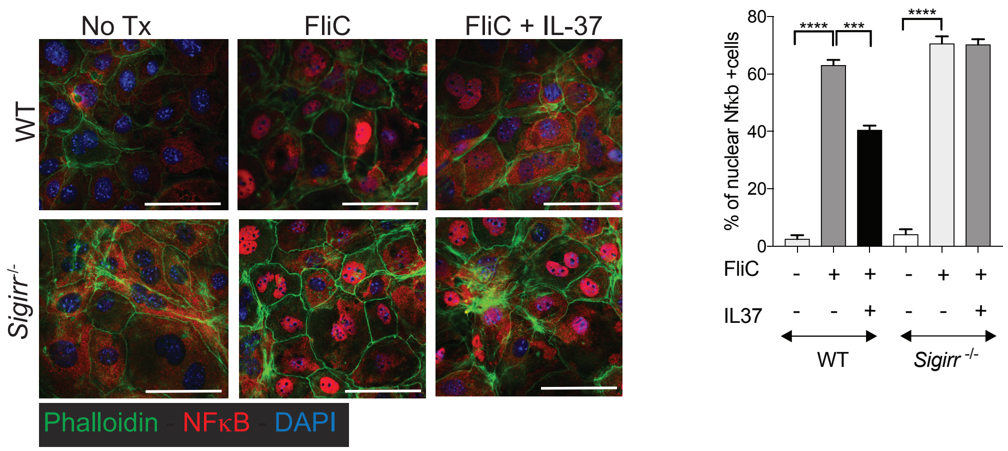

Figure S3: IL-37 reduces FliC induced Nfkb activation in mouse organoids. Immunostaining against Nfκb (red), Actin (Phalloidin - green) and DAPI (blue) of 2Ð mono-layer after 30 minutes of stimulation with FliC with or without IL-37 (left). Counts of Nfκb positive nuclei from immunostaining (Right). Mean and SEM are indicated from n= 4 mouse organoid lines derived from each genotype. All data shown are representative of at least 3 independent experiments.

Figure S4

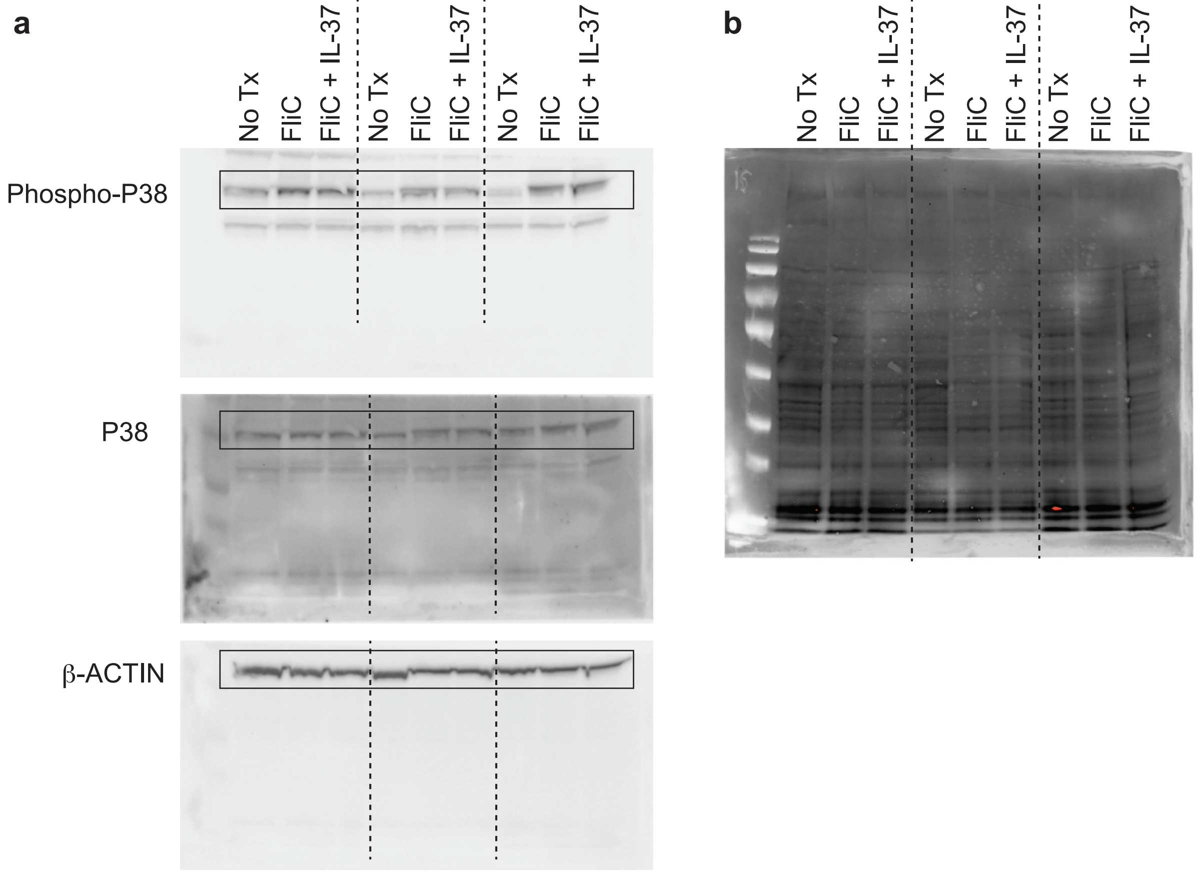

Figure S4: IL-37 does not reduce p38 activation in human colonoids grown in regular media. **a)** Representative immunoblot detecting phospho and total p38, in human colonoids grown in regular organoid media untreated (No Tx), or after 30 minutes of stimulation with FliC with, or without IL-37. Shown here is the whole PVDF membrane (cut into several pieces), cropped bands of interest (included in the black rectangle) are shown in Figure 2e. Dashed line separates organoid data derived from 3 different patients. **b)** Total protein stain of whole PVDF membrane (used for Fig.2e) additionally confirming comparable protein loading in all lanes.

Figure S5

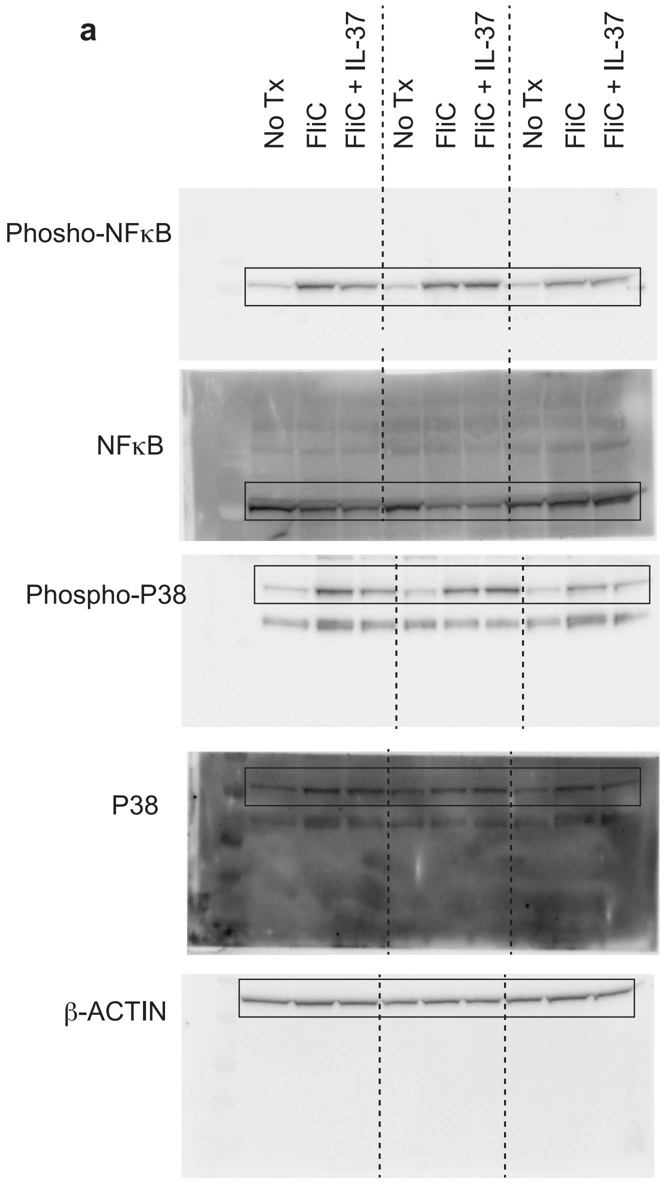

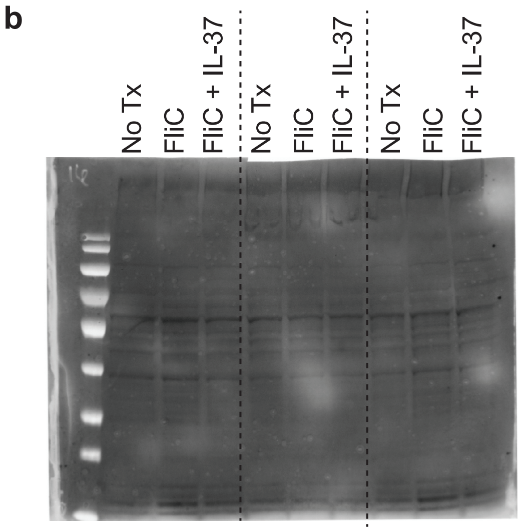

Figure S5: IL-37 reduces FliC induced NFkB and p38 activation in human colonoids.
a) Representative immunoblot detecting phospho and total p38, in human colonoids grown in (No SB202) media untreated (No Tx), or after 30 minutes of stimulation with FliC with or without IL-37. Shown here is the whole PVDF membrane (cut into several pieces), cropped bands of interest (included in the black rectangle) are shown in Figure 3e. Dash line separate organoid derived from 3 different patients. **b)** Total protein stain of whole PVDF membrane (used for Fig.3e) additionally confirming comparable protein loading in all lanes.

Figure S6

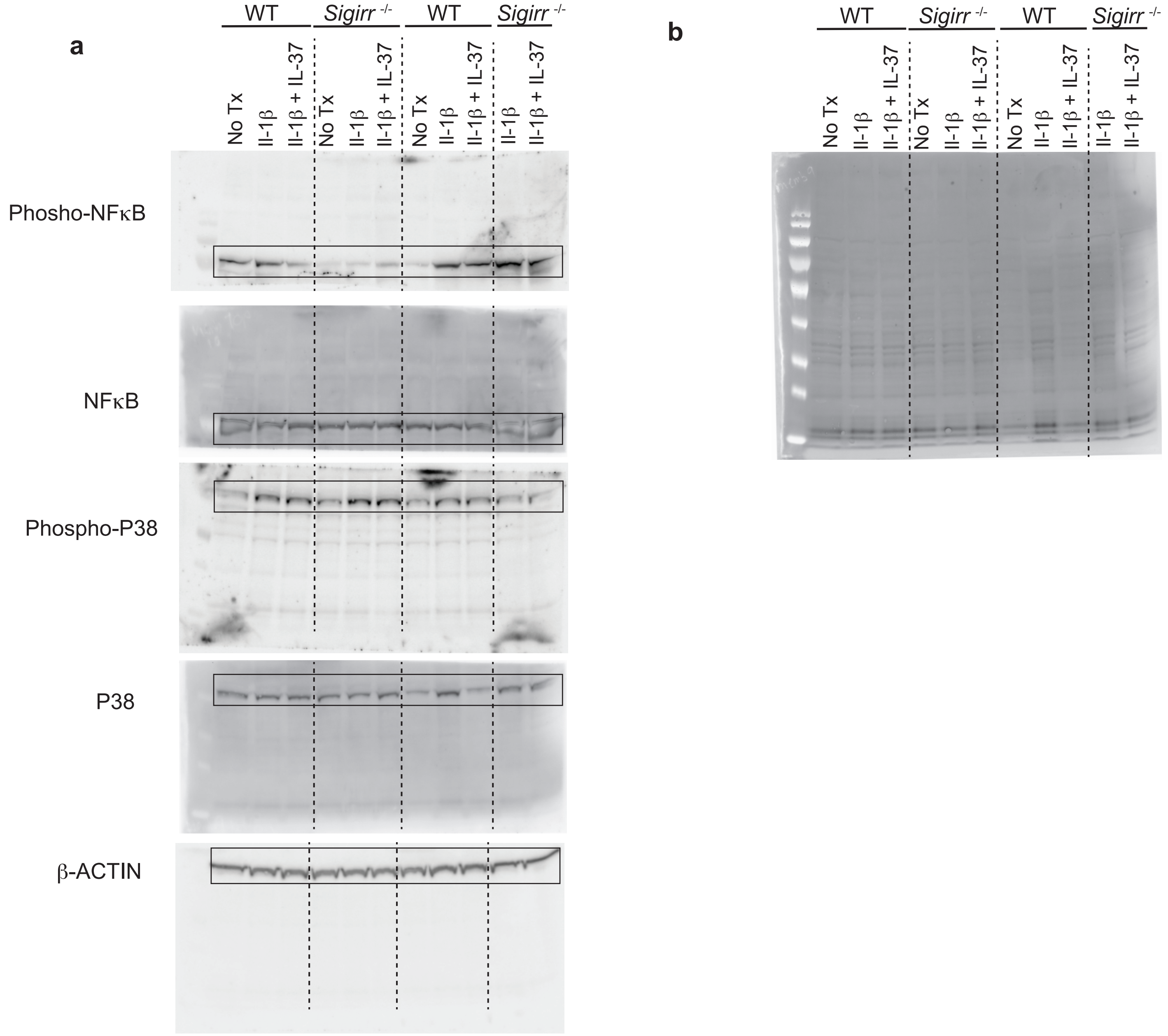

Figure S6: IL-37 suppression of innate responses in mouse organoids is Sigirr dependent. **a)** Representative immunoblot detecting phospho and total p38, in mouse colonoids untreated (No Tx), or after 30 minutes of stimulation with Il-1β with or without IL-37. Shown here is the whole PVDF membrane (cut into several pieces), the cropped bands of interest (included in the black rectangle) are shown in Figure 5f. The dashed line separates organoid derived from different mice. **b)** Total protein stain of whole PVDF membrane (used for Fig.5f) confirms comparable protein loading in all lanes.
